# Egg recognition: The importance of quantifying multiple repeatable features as visual identity signals

**DOI:** 10.1371/journal.pone.0248021

**Published:** 2021-03-04

**Authors:** Jesús Gómez, Oscar Gordo, Piotr Minias

**Affiliations:** 1 Independent researcher, Spain; 2 Catalan Ornithological Institute, Barcelona, Spain; 3 Department of Biodiversity Studies and Bioeducation, Faculty of Biology and Environmental Protection, University of Łódź, Łódź, Poland; Universita degli Studi di Perugia, ITALY

## Abstract

Brood parasitized and/or colonial birds use egg features as visual identity signals, which allow parents to recognize their own eggs and avoid paying fitness costs of misdirecting their care to others’ offspring. However, the mechanisms of egg recognition and discrimination are poorly understood. Most studies have put their focus on individual abilities to carry out these behavioural tasks, while less attention has been paid to the egg and how its signals may evolve to enhance its identification. We used 92 clutches (460 eggs) of the Eurasian coot *Fulica atra* to test whether eggs could be correctly classified into their corresponding clutches based only on their external appearance. Using SpotEgg, we characterized the eggs in 27 variables of colour, spottiness, shape and size from calibrated digital images. Then, we used these variables in a supervised machine learning algorithm for multi-class egg classification, where each egg was classified to the best matched clutch out of 92 studied clutches. The best model with all 27 explanatory variables assigned correctly 53.3% (CI = 42.6–63.7%) of eggs of the test-set, greatly exceeding the probability to classify the eggs by chance (1/92, 1.1%). This finding supports the hypothesis that eggs have visual identity signals in their phenotypes. Simplified models with fewer explanatory variables (10 or 15) showed lesser classification ability than full models, suggesting that birds may use multiple traits for egg recognition. Therefore, egg phenotypes should be assessed in their full complexity, including colour, patterning, shape and size. Most important variables for classification were those with the highest intraclutch correlation, demonstrating that individual recognition traits are repeatable. Algorithm classification performance improved by each extra training egg added to the model. Thus, repetition of egg design within a clutch would reinforce signals and would help females to create an internal template for true recognition of their own eggs. In conclusion, our novel approach based on machine learning provided important insights on how signallers broadcast their specific signature cues to enhance their recognisability.

## Introduction

The ability to identify other individuals is an essential cognitive skill of animals. Identification is important because it is necessary to recognize mates [1], own offspring [2, 3], competitors [4], predators [5] or preys [6], among other animal interactions [7, 8]. There are a great variety of mechanisms to identify other individuals according to their unique chemical (e.g., odour and taste) or physical (e.g., sounds and appearance) features [9]. Birds rely mainly on their visual and auditory sense to collect information about their environment [10–12] (but see [13, 14]). There is substantial evidence on the ability of birds to discriminate voices at individual level [1–4, 15–19], while the mechanisms underlying individual visual recognition are still poorly understood [1, 20–22]. Recognition processes associated with brood parasitism stand out as an exception, since evolutionary ecologists have paid great attention to the arms race between hosts and their brood parasites as a classical model of coevolution [23]. Due to the fitness costs paid by parasitized hosts, their ability to identify their own progeny and discriminate it from the parasite confers an obvious adaptive advantage [24–27].

Offspring recognition is also critical for colonial breeding birds [2, 28–32]. This task may be sometimes challenging, as nests can be placed close to each other in huge and dense colonies on uniform substrates, where birds may easily make mistakes in nest recognition and misdirect their parental care to the broods of conspecifics. Gaston et al. [33] argued that murres (*Uria* spp.) recognized and retrieved their own eggs more than foreign eggs, although these authors did not provided information on egg similarity. Recently, Hauber et al. [30, 34] showed that females laid eggs with repeatable patterning in different breeding attempts by controlling the physiochemical properties of the eggshell. These researchers concluded that such adaptive mechanism would help murres to recognize eggs by fixing an internal template of their own eggs. Murres, consequently, would not need to learn again their own egg patterns in future breeding attempts. In addition to the potential confusion problems, colonial birds often suffer from conspecific brood parasitism [27, 35–37], which adds another selective pressure for the evolution of reliable egg recognition mechanisms [26, 38].

Most studies on brood parasitism have put the focus on the ability of hosts (i.e., the signal receiver) for individual recognition and their subsequent egg rejection behaviour [39, 40]. To study this behaviour, an essential step is to characterize egg phenotypes, since host decisions depend on them [26, 41–44]. Traditionally, eggs have been characterized simplistically by one or a few phenotypic features, often focussing exclusively on eggshell coloration, which has been usually quantified by punctual measurements with spectrophotometers (e.g., [45–47]). This approach may be suitable for plain eggs, but it appears clearly insufficient for species with complex eggshell patterns (speckles, spots and patches). In these cases, a *de visu* categorization of the pattern has been a habitual approach [43, 45, 48–50], but it appears an arbitrary method subjected to biases by observer’s perception [51–54]. For this reason, digital pictures with a posterior objective image processing have gained popularity during the last decade as the most suitable way to quantify complex egg phenotypes [52]. However, there is no consensus on how to analyse and quantify images yet. Some authors employed available tools in commercial software, such as Photoshop, to determine colours and proportion of eggshell covered by marks (e.g., [30, 55, 56]). This approach can circumvent the problems of subjective *de visu* quantification in some cases, but suffers from a number of drawbacks due to its simplicity, as usually requires a handcraft image processing, subsamples small areas of the eggshell, and does not provide synthetic variables for the entire egg phenotype. Thus, several software packages have been designed to deal specifically with processing of complex egg images [21, 52, 57, 58]. Despite of this panoply of powerful tools, their application to study individual egg recognition has been quite limited yet (e.g., [59, 60]). The ability of these tools to detect and quantify patterns of variation undetectable to human eye seems suitable for the study of egg phenotype variation and shed light into how birds are able to recognize their own eggs.

The aim of our study was to investigate which egg phenotype features can be a signal for its individual recognition. For this purpose, we characterised egg phenotypic traits using SpotEgg [58] and evaluated which of these traits presented higher intraclutch correlation coefficient (hereafter, ICC). ICC has been widely used to quantify the degree of resemblance of the eggs belonging to the same clutch [30, 55, 60, 61]. Then, we used a supervised machine learning algorithm to determine whether or not eggs from the same clutch can be mathematically discriminated according to their features. Due to our limited understanding of the cognitive mechanisms used by the receiver (i.e., the bird [21]), we adopted an approach from the signaller (i.e., the egg) perspective [62], focusing our attention on egg traits, their patterns of variation, and how they can be objectively discriminated. We expected a positive correlation of the ICC with the most important egg features chosen by the classifier algorithm. This positive correlation was expected because identity signals of eggs should be condition independent, highly variable among individuals, and repeatable over individual’s lifetime (i.e., genetically determined) [21, 61, 63].

**Fig 1:**
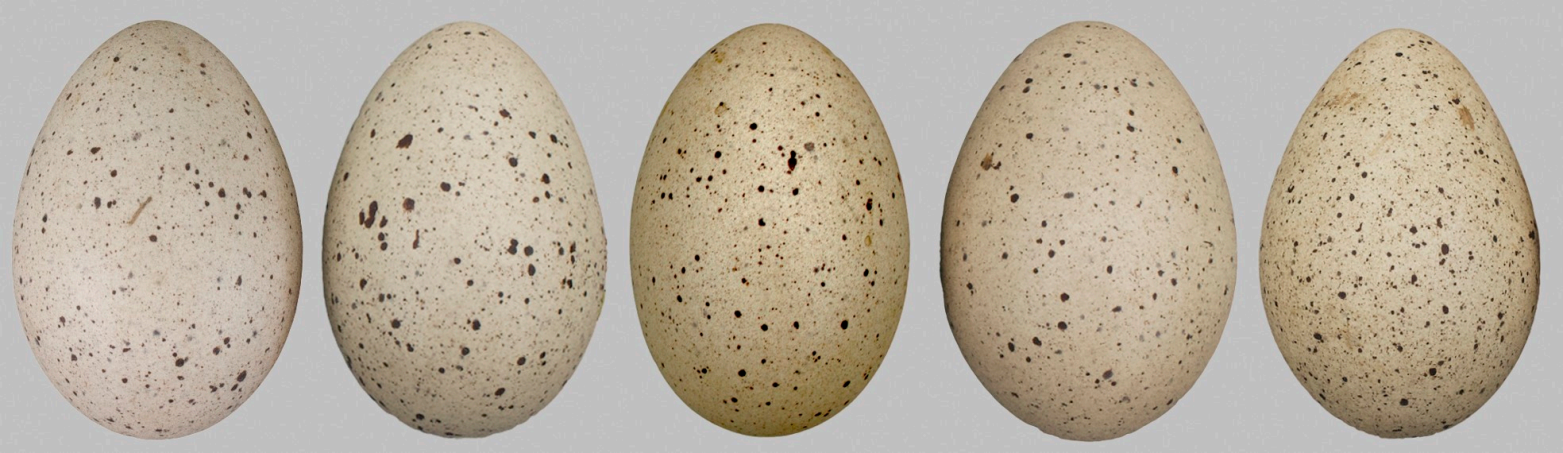
Phenotype variability in Eurasian coot eggs. Five example eggs from different clutches. Differences in colour, spottiness, shape and size are very subtle and hardly perceptible by human eye.

We used clutches of the Eurasian coot *Fulica atra*, a common waterbird with protoporphyrin spotted eggs (Fig 1; [64]), as a model species to test our hypotheses. The Eurasian coot is neither a high-density colonial bird or a regular host of interspecific brood parasites, so females are not apparently subjected to a high selective pressure to lay individually distinctive eggs. Nevertheless, as in other related rail species [26, 38, 49, 65–68], conspecific brood parasitism seems fairly common in this species [69, 70], and thus Eurasian coots may actually be under some evolutionary pressure to recognize own eggs [27].

## Materials and methods

### Study sites and image collection

We studied coots at three nesting sites in central Poland: Łódź (51° 46’ N, 19° 28’ E), Sarnów (51° 51’ N, 19° 07’ E), and Żeromin (51° 37’ N, 19° 37’ E). Łódź was an urban area (large city with ca. 700.000 inhabitants), while the other two sites were fishponds located in rural areas. At each site we searched for coot nests in the available nesting habitat (mostly reeds) and, if found at the laying stage, we monitored the clutches until completion.

In our study population, the median clutch size was seven eggs with the lower and upper quartiles of six and nine eggs, respectively. The mean egg volume was 35.59 ± 0.15 (SE) cm^3^. Most breeding pairs had only one breeding attempt (second clutches were laid by ca. 3% of pairs), although repeated clutches were often recorded following breeding failures. Incubation period lasted 21–24 days and adults cared for young for up to two months. In our study population, laying started at the end of March and lasted until the beginning of July, although most first clutches were laid before the end of May.

To assess eggshell phenotype, we randomly selected five eggs per clutch and took a photograph of them in 2017 and 2018 (from March to July). In total, we photographed 92 clutches (460 eggs), most of which originated from the urban population of Łódź (64 clutches). Each egg was cleaned with a cloth and all vegetation particles were removed from shell surface. All photographs were taken following recommendations by Gómez and Liñán-Cembrano [58]: eggs were placed on a grey standard (Lastolite Ezybalance, 30 cm, 18% reflectance), next to a standard colour chart (ColorChecker Passport, USA X-Rite), and photographed from a distance of approximately 30 cm. The photographs were taken in RAW format (5202x3464 pixels) using a Canon EOS 6D digital camera. Exposure settings were adjusted accordingly with lightning conditions, yet the ISO value was set constant at 400. After collecting photographs, all eggs were returned immediately to their nests.

### Ethics statement

The study was conducted in accordance with the current laws of Poland, where it was performed under the permission of the Local Bioethical Commission for Experiments on Animals in Łódź.

### Image processing

Conventional digital cameras employ a non-linear radiometric transfer function to produce visually appealing images at the expense of a reduction of the contrast in the areas where contrast is originally low. In consequence, images need to be linearized with respect to irradiance to obtain meaningful reflectance results [58]. For this reason, we transformed images into equivalent reflectance images based on the information from the pixels and using the grey scale of the standard colour chart. Once the spottiness was detected and spot images were created, we estimated the percentage of the eggshell covered by spots (degree of spottiness) plus other variables related with the spottiness (Table 1). Spot detection for each picture was executed using an image-processing parameterised algorithm that basically relies on defining optimised spatially variant thresholds to segment spots from the background. After that, we obtained the red, green and blue colour channels for the whole eggshell as well as for the spots and the background separately. The volume and other size-related egg variables were also calculated (Table 1). All image processing and variable calculations were performed with the free software SpotEgg [58], which was originally run through the Matlab (MathWorks, Natick, MA, USA), but now it can be executed via an.exe file (for detailed information on how to download the version 1.0 of the program visit: https://guslicem.wixsite.com/spotegg). We used the background colour constancy option.

**Table 1:** List and description of the 27 variables calculated by SpotEgg software to characterise egg phenotype.

Variable	Description
**Volume**	Volume from integrating your Region of Interest (RoI) as a revolving surface shape generator (mm^3^).
**Area**	Area from integrating your RoI as a revolving surface shape generator (mm^2^).
**Length**	Major axis length of the ellipse that has the same second order moments as your RoI (mm).
**Width**	Minor axis length of the ellipse that has the same second order moments as your RoI (mm).
**NumSpots**	Number of detected Spots in the RoI.
**TotAreaSpots**	% of RoI covered by spots.
**AvgSpotSize**	Mean spot size (%) for the spots in this RoI.
**AvgEccentricity**	Mean eccentricity for the spots in the RoI. The eccentricity is the ratio of the distance between the foci of the ellipse that has the same second order moments as your RoI and its major axis length.
**FractalDim**	Fractal dimension for the spottiness pattern in the RoI.
**TotalR**	Mean equivalent reflectance in the camera’s Red channel for all the pixels in the RoI.
**TotalG**	Mean equivalent reflectance in the camera’s Green channel for all the pixels in the RoI.
**TotalB**	Mean equivalent reflectance in the camera’s Blue channel for all the pixels in the RoI.
**SpotsR**	Mean equivalent reflectance in the camera’s Red channel for all the spots in the RoI.
**SpotsRSTD**	Standard deviation for the equivalent reflectance for the spots in the RoI in the camera’s Red channel.
**SpotsG**	Mean equivalent reflectance in the camera’s Green channel for all the spots in the RoI.
**SpotsGSTD**	Standard deviation for the equivalent reflectance for the spots in the RoI in the camera’s Green channel.
**SpotsB**	Mean equivalent reflectance in the camera’s Blue channel for all the spots in the RoI.
**SpotsBSTD**	Standard deviation for the equivalent reflectance for the spots in the RoI in the camera’s Blue channel.
**BackGroundR**	Mean equivalent reflectance in the camera’s Red channel for the background in the RoI.
**BackGroundRSTD**	Standard deviation for the equivalent reflectance for the background in the RoI in the camera’s Red channel.
**BackGroundG**	Mean equivalent reflectance in the camera’s Green channel for the background in the RoI.
**BackGroundGSTD**	Standard deviation for the equivalent reflectance for the background in the RoI in the camera’s Green channel.
**BackGroundB**	Mean equivalent reflectance in the camera’s Blue channel for the background in the RoI.
**BackGroundBSTD**	Standard deviation for the equivalent reflectance for the background in the RoI in the camera’s Blue channel.
**Per_vs_Area**	Mean perimeter/area ratio for the spots on the RoI. It gives an insight on the average shape roughness of spots.
**EquivAxisL**	Length (from 0—pointy end- to 1) across the longitudinal axis of the spots when one can consider that all the spots are concentrated. It is a kind of longitudinal center of mass for spottiness.
**Max_Spot_Con**	Length (from 0—pointy end- to 1) across the longitudinal axis of the spots when the maximum spottiness occurs.

### Statistical analyses

We used Support Vector Machines (SVMs) as a proxy of the perception of any observer (e.g., a bird female). SVMs are supervised algorithms which sort data into categories (classifiers). SVMs use multidimensional surfaces to define the relationship between features and outcomes. In other words, they use a boundary called a hyperplane to split data into groups of similar class values [71, 72].

To evaluate the classification performance of different models we took into account two aspects of the SVM: the number of explanatory variables and the number of eggs used during model training. On the one hand, we compared SVMs with a different number of explanatory variables. As it has been suggested that SVMs might be possibly sensitive to multicollinearity [73], we checked model performance without the most correlated variables to avoid potential overfitting and enhance the generality of the classifier. For this purpose, we set two arbitrary thresholds from the Spearman’s correlation among variables (S1 Fig): 0.97 (extremely correlated) and 0.75 (highly correlated). For each pair of correlated variables, we left those more relevant *a priori*. For example, we kept the green channel instead of the correlated red or blue channels because of the greater importance of green wavelengths in avian vision [13]. In the reduced set of variables with *r*_s_ < 0.97, we removed 12 out of the 27 variables (Area, TotalR, TotalG, TotalB, SpotsR, SpotsB, BackgroundR, BackgroundB, SpotsRSTD, SpotsBSTD, BackGroundBSTD and BackGroundRSTD). In the reduced set of variables with *r*_s_ < 0.75, we removed five more variables (Volume, TotAreaSpots, FractalDim, SpotsGSTD, and BackGroundGSTD). We kept in the latter subset both SpotsG and BackGroundG despite their relatively strong correlation (*r*_s_ = 0.84), to have at least one variable to characterize the coloration of the spots and the background. Therefore, we obtained three different sets of models with 27 (i.e., the total number of variables calculated by SpotEgg), 15 (variables with *r*_s_ > 0.97 removed) and 10 (variables with *r*_s_ > 0.75 removed, but see the exceptions above) explanatory variables.

On the other hand, we run four SVMs that were trained with 1, 2, 3 and 4 eggs, respectively, in each one of the three previous sets of explanatory variables. This comparison might help to understand how repeatability of an egg design within a clutch may reinforce the message sent by the signaller.

We randomly selected the training-set and the test-set for each triplet of models. We applied a repeated (N = 3) cross-validation of K = 10 for the test-sets and a radial basis kernel Gaussian algorithm for all SVMs. Cost value was optimised to 15. Because this is a multi-class classification problem, the probability to assign one egg to its correct label (i.e., its own clutch) is much lower than to assign it to one of the remaining 91 labels. Consequently, the probability to assign correctly by chance one egg to a nest (N = 92) was 1/92 (Random accuracy = 1.1%). We used the accuracy as a measurement of performance of the classification task. Accuracy was calculated as the overall success of the labelling process of each test-set. On the other hand, the importance of each variable in the classifier was calculated averaging the results of 50 SVMs run with 27 variables and trained with 4 eggs to get more robust estimations.

Finally, we used ICC to estimate the repeatability of eggshell features within clutches [74]. We estimated the real ICC and a simulated ICC by replacing randomly one egg of each clutch by another egg from another clutch. The latter ICC aims to assess how the ICC changes in simulated nests with one parasitic egg. If eggs resemble each other within clutches more than among clutches, as we expected, the simulated ICC will be lower than the real ICC. We used 1,000 parametric bootstraps for interval estimation and 10 permutations to calculate p-values.

P-values were adjusted for multiple testing using the Benjamini and Hochberg [75] correction. All statistical analyses were carried out in R statistical software version 3.6.3 [76]. The package used for SVM analyses was kernlab version 0.9–29 [77], which implements a one-against-all SVM. The package used for ICC calculations was rptR version 0.9.22 [78]. Significance level was set at α = 0.05.

## Results

### Support vector machines

The best classification was achieved when the algorithm used the 27 variables calculated by SpotEgg and was trained with 4 eggs per clutch (Table 2). It classified correctly *de novo* 49 of 92 eggs (accuracy = 53.3%) from the test-set, which greatly exceeded the random accuracy (1.1%) in this multi-class classification problem.

**Table 2:** Comparison of the accuracy (%) reached by the 12 Support Vector Machines run using a different number of training eggs and sets of explanatory variables. The percentage of egg classification by random (random accuracy) was 1.1%.

Training eggs	No. variables	Accuracy	95% CI
1	10	12.8	[9.5–16.6]
	15	18.2	[14.4–22.5]
	27	19.6	[15.6–24.0]
2	10	22.5	[17.7–27.8]
	15	30.1	[24.7–35.8]
	27	35.5	[29.9–41.5]
3	10	25.5	[19.4–32.5]
	15	34.8	[28.0–42.1]
	27	36.4	[29.4–43.8]
4	10	39.1	[29.1–49.9]
	15	46.7	[36.2–57.4]
** **	27	**53.3**	**[42.6–63.7]**

The best model is highlighted in bold. The 95% confident intervals (CI) for the accuracy are shown.

Accuracy of SVMs increased by adding either more training eggs and/or more variables (Table 2). A single training egg produced relatively poor classification results (accuracy 12.8–19.6%), which were notably enhanced by adding the second training egg (accuracy 22.5–35.5%; Table 2). Adding a third training egg did not improve much the results compared to the SVMs with two eggs, while the addition of a fourth training egg made the SVMs notably better. The greater improvement was achieved during transition from 10 to 15 variables, while the transition from 15 to 27 increased the accuracy to a lesser extent (Table 2).

**Fig 2:**
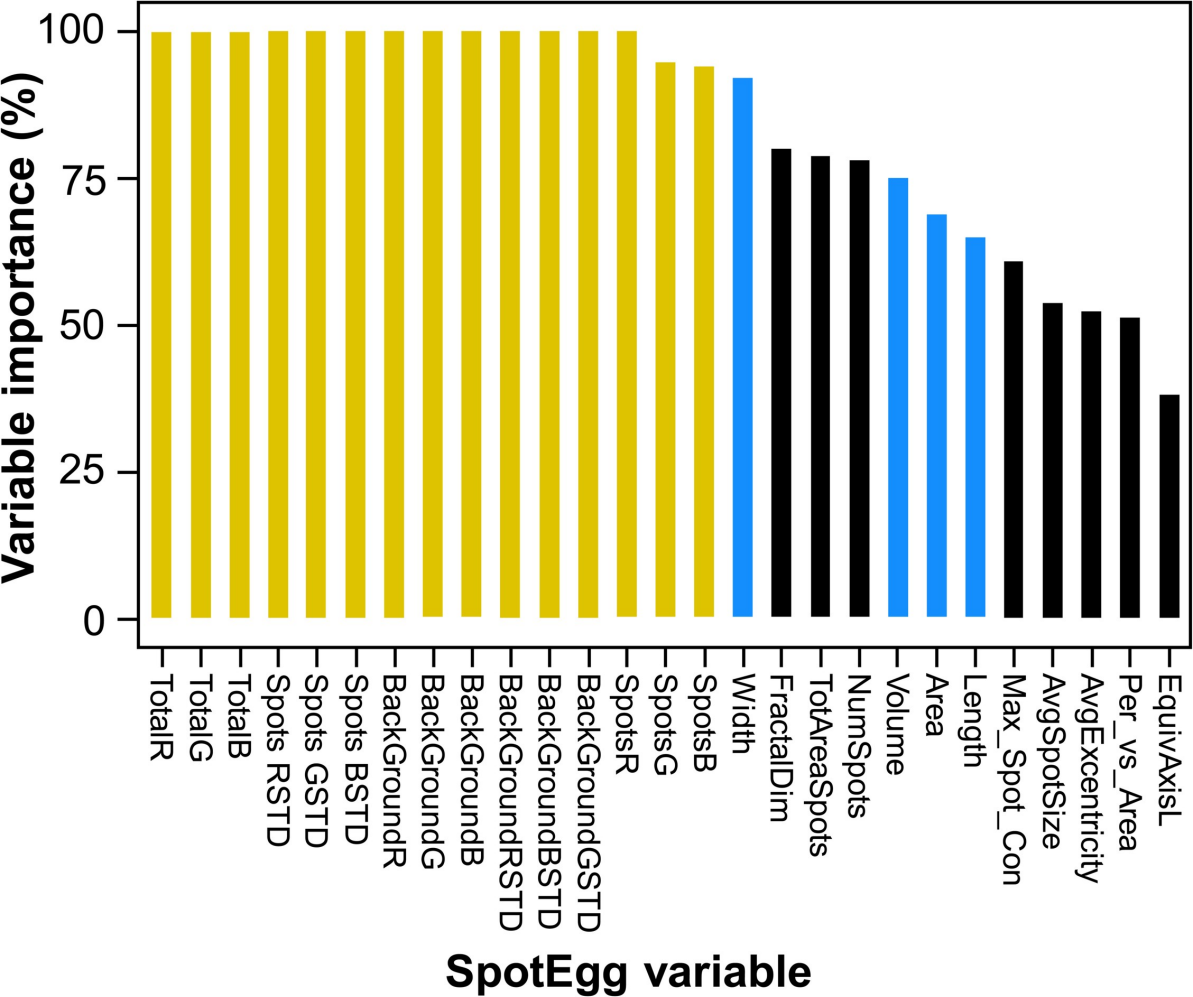
Variable importance (%) in the best Support Vector Machine (SVM). Best SVM contained 27 variables and 4 training eggs. Colours group related egg features (ochre: coloration; blue: size and shape; black: spottiness).

Eggshell coloration variables had the highest importance for classification, followed by those related to some characteristics of the spottiness, egg shape and size, yet the less important features were those related to the distribution of the spottiness across the eggshell (Fig 2). As predicted, variable importance showed a strong positive correlation with the real ICC values (*r*_s_ = 0.814, p < 0.001).

### Intraclutch correlation coefficients (ICC)

Most variables had a high repeatability within clutches (Table 3). Actually, only five variables showed poor repeatability (ICC < 0.5; Table 3), yet they were significantly greater than zero, except for Max_Spot_Con (P = 0.053). However, as we expected, ICC dropped drastically under the simulation of a parasitic egg added to the clutch (Table 3). In fact, on average, repeatability was 0.283 (SD = 0.094) lower in the clutches with a simulated parasitic egg than in the real ICC values.

**Table 3:** Real and simulated Intraclutch Correlation Coefficients (ICC) for egg variables calculated by SpotEgg.

Variable	Real ICC [CI]	Simulated ICC [CI]
**Volume**	0.704 [0.613–0.771]	0.350 [0.252–0.446]
**Area**	0.701 [0.615–0.764]	0.344 [0.242–0.436]
**Length**	0.667 [0.573–0.738]	0.328 [0.222–0.420]
**Width**	0.682 [0.602–0.748]	0.346 [0.241–0.441]
**NumSpots**	0.652 [0.561–0.721]	0.443 [0.340–0.538]
**TotAreaSpots**	0.613 [0.513–0.691]	0.394 [0.288–0.491]
**AvgSpotSize**	0.432 [0.327–0.520]	0.304 [0.206–0.406]
**AvgExcentricity**	0.214 [0.117–0.310]	0.142 [0.050–0.223]
**FractalDim**	0.620 [0.530–0.694]	0.393 [0.284–0.480]
**TotalR**	0.810 [0.749–0.852]	0.458 [0.358–0.549]
**TotalG**	0.807 [0.748–0.853]	0.463 [0.359–0.555]
**TotalB**	0.801 [0.728–0.845]	0.466 [0.365–0.557]
**SpotsR**	0.689 [0.602–0.755]	0.391 [0.293–0.488]
**SpotsRSTD**	0.778 [0.708–0.828]	0.440 [0.332–0.526]
**SpotsG**	0.666 [0.573–0.735]	0.377 [0.280–0.474]
**SpotsGSTD**	0.779 [0.709–0.827]	0.447 [0.338–0.543]
**SpotsB**	0.670 [0.582–0.744]	0.385 [0.282–0.475]
**SpotsBSTD**	0.782 [0.714–0.836]	0.454 [0.343–0.548]
**BackGroundR**	0.812 [0.746–0.856]	0.461 [0.356–0.549]
**BackGroundRSTD**	0.885 [0.844–0.913]	0.515 [0.417–0.597]
**BackGroundG**	0.809 [0.743–0.853]	0.465 [0.353–0.554]
**BackGroundGSTD**	0.868 [0.820–0.902]	0.509 [0.401–0.594]
**BackGroundB**	0.803 [0.737–0.849]	0.468 [0.362–0.560]
**BackGroundBSTD**	0.850 [0.801–0.885]	0.493 [0.385–0.583]
**Per_vs_Area**	0.439 [0.334–0.528]	0.247 [0.148–0.341]
**EquivAxisL**	0.232 [0.137–0.325]	0.083 [0.010–0.171]
**Max_Spot_Con**	0.058 [0.000–0.133]	0.018 [0.000–0.085]

See Table 1 for variable description. The 95% confident intervals (CI) are shown.

## Discussion

The machine learning algorithm provided an objective way to correctly classify more than half of the eggs into their original clutches based exclusively on their external features. This is a good achievement taking into account that the algorithm faced a multi-class classification problem where probability of classification success by chance was only ca. 1%. This achievement also appears good due to the extraordinary resemblance of eggs among clutches of the studied Eurasian coots, at least, from a human eye perspective (see Fig 1). In fact, this accuracy level was similar to the discriminatory ability found in several bird species towards parasitic eggs [23, 25, 42, 43]. Therefore, coupling image processing software, such as SpotEgg, with advanced classification algorithms may help to decipher the non-patent visual identity signals of eggs. These visual identity signals of the egg phenotype can be used for its individual recognition, which is a great adaptive advantage in the context of brood parasitism [21, 26, 43, 59, 79] and colonial breeding [2, 28, 32]. For this reason, it is not surprising that eggs have developed reliable identity signals in bird species exposed to these evolutionary scenarios.

As we predicted, we found a strong positive correlation between intraclutch correlation (ICC) values and the variable importance during the classification procedure. Therefore, the algorithm relied primarily on the most repeatable eggshell features to achieve individual recognition. This finding perfectly agrees with the theoretical prediction that individual signals should be replicable or repeatable [21, 63]. Several studies demonstrated that females of some bird species lay eggs with the same colour and patterning (i.e., they are highly repeatable) during their whole life [30, 42, 43, 60, 80], but here, to the best of our knowledge, we provided the first demonstration that such repeatability may be indeed linked to egg individual recognition.

The most important variables for individual visual recognition were related to the coloration features of the egg, followed by some variables related to the spottiness and to the size or shape of the egg. This order of importance agrees with previous studies in some passerine species, such as the Tawny-flanked prinia *Prinia subflava* [81] or the Village weaverbird *Ploceus cucullatus* [42, 43]. In contrast, egg discrimination in the Common quail *Coturnix coturnix* [55, 80] and the Goldeneye *Bucephala clangula* [82, 83] relied on the eggshell patterning or egg morphology, respectively. Such discrepancies among studies were expected under an evolutionary perspective, since there is no single evolutionary pathway to develop individual egg recognition. Each species has phenotypic traits more prone to evolve towards identity signals than others, as egg phenotypic traits may be also constrained by other important functions [79, 84, 85], such as camouflage [80, 86], protection against radiation [87], or flight biomechanics [88]. This fact may explain why in some species most variation among eggs arises in colour [42, 43, 46, 81], while in others the eggs primarily vary in shell marks [28, 55, 80], shape and size [82, 83], or a combination of different traits [21, 30, 60, 89]. Theory predicts that individual recognition traits must be independent [63], so they can maximize the diversity of combinations among individuals and contain the greatest amount of information [32, 34, 81]. Such independence among traits guarantees free evolutionary trajectories for each trait and this fact would explain the variety of phenotypic solutions for egg recognition found in nature [21, 79].

We used a large array of variables obtained automatically by the image processing software SpotEgg [58] to describe objectively the egg phenotype in its full complexity. Not surprisingly, some of these variables were strongly inter-correlated. For instance, the average size of spots is expected to decrease as their number increases for a certain eggshell area (see S1 Fig). On the other hand, the colour of the egg results from the combination of the spots and background colours and, thus, the full egg colouration will, by definition, be correlated with the colouration of its components. Despite of these redundancies in the information encoded by our variables, classification accuracy of the algorithm dropped when we removed the most correlated variables. This fact was unexpected, since variables removed from the model did not contain much unique information. This result would demonstrate that egg phenotype is highly complex and should be quantified in all its possible variation axes of colouration, spot pattern, shape and size, even if they seem redundant. In this sense, image processing by algorithms that allow a simultaneous quantification of multiple traits [21, 32, 52, 57, 58, 81] should be used in future studies of the egg phenotype. Traditional approaches that focused on a single trait or one trait category (e.g., colour, [46, 47, 56, 61]; spots, [48, 50]; or shape and/or size [70, 82, 83]), especially when estimated *de visu* [26, 48–50], may be insufficient to appropriately quantify information that eggs convey, limiting our ability to understand the evolution of egg individual signals and the mechanisms by which birds interpret them. For example, we quantified colouration by 15 different variables that separately described spots and background, which is not always feasible using punctual measures provided by spectrophotometers [52, 58]. Spottiness has usually been quantified just as the percentage of the surface of the egg covered by marks (e.g., [30, 55]). Once again, simple approaches may often lead to simplistic conclusions and, thus, we strongly encourage to use mathematical algorithms designed to objectively parameterize both quantitative (number, size) and qualitative (distribution, shape) aspects of egg spottiness (e.g., [21, 52, 57]). For example, Gómez et al. [90] recommended spottiness fractal dimension as one of the key spottiness parameters, as it is well correlated with the amount of protoporphyrin pigments in the shell (see also [34]). Therefore, only by an appropriate mathematical quantification of the complexity of spot patterns, we may successfully reveal the identity signals encrypted in eggshells. Finally, we also encourage researchers to quantify UV egg reflectance whenever possible because it may improve egg recognition, as birds have a tetrachromatic colour space [12].

Egg size and shape played a medium role for determination of egg identity in our study species. This result was to some extent unexpected, as egg size is frequently condition dependent (best condition females produce bigger eggs) and, in consequence, it was not predicted to be more useful for individual recognition than other spottiness-related features [62, 63]. However, egg features related with shape and size had medium-to-high ICC in our model species, which may explain why those features were moderately important for the classification. On the other hand, there is some controversy in the scientific literature about the role played by the shape and size of the eggs in the rejection rates, which may be dependent on the species studied. Victoria [42] found that Village weaverbirds did not reject foreign eggs, even if they were 20% bigger than their own eggs, as long as they showed similar colour and maculation pattern, the traits actually used for own egg recognition. Rothstein [40], during a series of experiments on several songbird species, also found that egg volume was not used to recognize and reject alien eggs, yet he also found the opposite in American robins *Turdus migratorious*. However, egg shape and size has already proved useful in detection of conspecific (e.g., [26, 82, 83]) or non-conspecific (e.g., [91]) brood parasitism by birds. Cheng et al. [69] showed that by using only variability of length and diameter of eggs within clutches of the Eurasian coot, they could determine whether or not a clutch was parasitized. Paradoxically, egg dimension was not sufficiently accurate to identify the parasite egg in most clutches and, consequently, individual egg recognition was not possible relying only on egg shape and size. Although, theoretically, egg size and shape should not be relevant for individual recognition as they are condition dependent [62, 63], they may show some degree of consistency within a single clutch. As clutches are laid in a short time frame (usually within a few days), one may expect that most of them would be produced under similar physiological condition of the female, they would receive a similar investment, and consequently they would have a similar size. Even if females lay eggs that vary in size and/or shape between first and second clutches or between consecutive breeding seasons (e.g., [30, 92]), it should not impact their ability to identify eggs from the same clutch. This is exactly what we found in our study and, for this reason, egg size and shape were more relevant than expected.

Algorithm classification performance improved with each extra egg added to train the SVM. Moreover, the accuracy did not show signs of flattening up to four training eggs, suggesting that the classifier could go beyond by using more than four training eggs. This would be feasible, as the Eurasian coot usually lays larger clutches than five eggs and thus, females have in natural conditions more information than our algorithm. The improvement of the algorithm with extra training eggs would suggest that sibling eggs act jointly to reinforce their signalling, supporting the true recognition hypothesis [38, 40–42]. The algorithm would create and improve an egg template for each clutch in an analogous process to learning found in some bird species [43, 80, 93, 94]. The more “observed” eggs by the algorithm (or female), the more accurate the template is, and consequently it should be able to better identify the eggs belonging to the same clutch. In our case, this resulted in a better classification performance, while in nature this ability could be translated into a better rejection of parasitic eggs [38, 41–44, 80].

Our machine learning classifier achieved unexpectedly good rates of accuracy with the Eurasian coot eggs, although it is not a species breeding in dense colonies neither a typical host of interspecific brood parasitism, the classical study models for egg individual recognition. These good rates of classification can be possible only if there is a higher variability among than within clutches, a typical evolutionary scenario where selection favoured the development of identity signals to distinguish own eggs. In fact, we found that intraclutch correlation in all phenotype traits dropped by adding a foreign egg to the clutches, supporting the hypothesis that eggs within a clutch resemble more than among clutches. It is known that the Eurasian coots suffers from conspecific brood parasitism [69, 70]. In a sister species, the American coot *Fulica americana*, it has been shown that such conspecific brood parasitism has similar fitness costs than those reported for songbirds most heavily parasitized by cuckoos [26]. Therefore, one should not be surprised to find mechanisms for individual recognition of eggs in the Eurasian coot, as a primary defensive weapon against conspecific parasite females [27]. We had no information about the current degree of conspecific brood parasitism in the study population, but it was expected to be low or even negligible. Most clutches used in this research were from an urban site, where pairs were scattered over small waterbodies in parkland areas. Many of these sites were occupied by a single breeding pair and in most cases, we did not observe non-breeding birds during the reproductive season. Thus, potential for conspecific brood parasitism was highly limited by bird distribution in our study population and clutches could hardly contain parasite eggs. This particular configuration of the breeding population distribution is quite different from the high breeding densities found in other studied populations [69, 70]. In the latter ones, birds would easily access neighbouring nests, which could enhance conspecific brood parasitism. Thus, it is possible that birds from our study populations may still preserve egg identity signals as a reminiscence of previous strongly selective scenarios of parasitism [21, 51, 79, 95; but see 45]. Therefore, our approach may be useful to reveal past or current selection processes leading to the evolution of individual egg recognition signals in the traditionally overlooked conspecific brood parasite systems [26, 27, 37, 38].

Although parasitism is a strong pressure for the development of individual recognition mechanisms, egg phenotype is usually subjected to other selective pressures (e.g., camouflage, thermoregulation, protection against radiation, microbes or rupture of the eggshell, etc. [56, 84–87, 96, 97]). These processes may indirectly lead females to produce personalized and recognizable patterns of colouration and spottiness on their eggs. For example, ground-nesting birds seem aware about the appearance of their eggs and lay them in a specific substrate to improve camouflage [86, 98]. If females specialize in or segregate by nesting substrates, this may lead to the appearance of different egg phenotypes among females, each one adapted to maximize camouflage and increase female fitness. Egg phenotype may also be affected be sexual selection [99]. Recently, Minias et al. [100] have found in the Eurasian coot that females in better condition and greater ornamental expression produced eggs with more spots, which may be a signal for males to increase their parental investment. This condition dependence contravenes the theoretical properties of any identity signal [62, 63], but, as explained previously, in our study context of single clutches, even this type of traits may work as identity signals. Actually, the number of spots was the third most important variable in the process of egg recognition among the variables employed to quantify maculation patterns (see Fig 2). A female coot in good condition would lay eggs with more spots and this characteristic of her eggs may help both to signal her quality to the male and to enhance distinctness from parasitic eggs laid by females in worse condition (with less spots).

Our approach based on machine learning aimed to be a proxy of the sensory and cognitive tasks of a bird (i.e., the receiver) challenged to identify its own eggs (i.e., the signallers). Interestingly, our algorithm achieved a similar discriminatory capacity to that shown by birds. For instance, Victoria [42] reported rejection rates <50% in most of their experiments with Village weaverbirds faced to discriminate against foreign spotted eggs. Lahti and Lahti [43] found rejection rates between 25 and 85% depending on the degree of difference between the parasitic egg and the own eggs in the same species. Øien et al. [25] found that only 38% of Reed warblers *Acrocephalus scirpaceus* realized that their nest had been parasitized by cuckoos *Cuculus canorus*. Finally, Lyon [23] reported that only 42.9% of parasitized females in American coots rejected at least one egg. Obviously, our approach does not allow to disentangle the complex neuro-physiological processes in the avian brain and visual systems of birds, as we focussed on the usually neglected signaller [62]. However, we have found important properties in the egg phenotype that may help to understand how signallers broadcast their specific signature cues to enhance their recognisability. First, birds would probably not use a single egg trait, such as colour, marks or size. They would probably assess the egg as a whole, as the more variables were included in the model, the better was its classification ability. Second, the egg features with higher intraclutch correlation were those more important for egg classification, demonstrating the theoretical prediction that individual recognition traits are repeatable or fixed. Third, repetition of a specific egg design in the same clutch would reinforce the overall message sent to the receiver (i.e., the parents), as this would help to create an internal template and true recognition. Fourth, labile traits, as those condition dependent, may also play a relevant role in egg recognition. As egg phenotype is an ephemeral signal (it works at most during the short time frame from laying to hatching), the physiological conditions of the female during the laying period may left indirectly a particular fingerprint in each clutch, which may help to increase the information encoded in the eggs and make clutches even more different and identifiable among females.

## Supporting information

S1 Fig Correlation matrix for the 27 explanatory variables obtained with SpotEgg. Variables were ordered using hierarchical clustering. Spearman’s correlation coefficients were coloured depending on their value following the scale plotted on the right.
(JPEG)

S1 Dataset Variables obtained from SpotEgg image processing. Each row is an image (egg). See Table 1 for variable description. A nest identifier to group sibling eggs is also available.
(TXT)
