# Supplementary figures and images for: Egg recognition: The importance of quantifying multiple repeatable features as visual identity signals
Source: PLoS One. 2021 Mar 4;16(3):e0248021. doi: 10.1371/journal.pone.0248021 (PMC7932075; doi:10.1371/journal.pone.0248021)

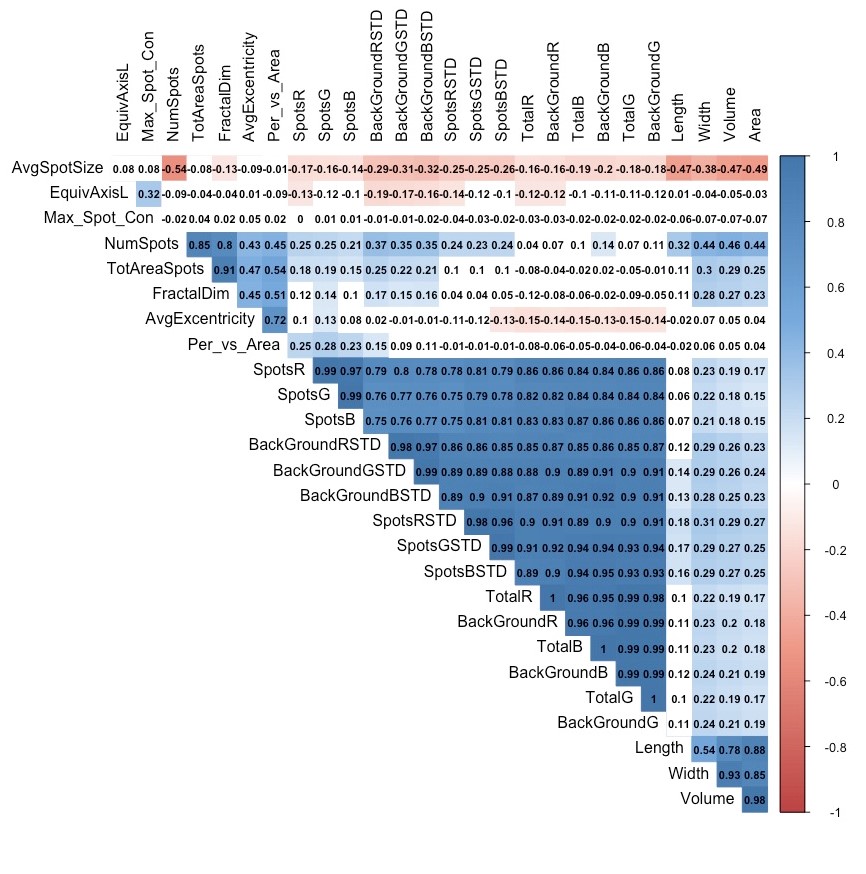

Supplement: S1 Fig — Variables were ordered using hierarchical clustering. Spearman’s correlation coefficients were coloured depending on their value following the scale plotted on the right. (JPEG) [file pone.0248021.s001.jpeg]
